# Can Routine Laparoscopy Help to Reduce the Rate of Explorative Laparotomies for Gastric Cancer? Laparoscopy in Gastric Cancer

**DOI:** 10.1155/DTE.6.125

**Published:** 2000

**Authors:** Gian Carlo Roviaro, Federico Varoli, Davide Sonnino, Ombretta Nucca, Gianni Rabughino, Alessandro Scarduelli

**Affiliations:** Department of General Surgery University of Milan Ospedale San Giuseppe FBF, Via San Vittore 12 Milano 20123 Italy

## Abstract

*1. Background* We developed this surgical protocol about performing intraoperative laparoscopy for staging in every patient affected by stomach cancer. Sensitivity and specificity of intraoperative laparoscopy are compared with conventional preoperative staging techniques.

*2. Methods* From January 1994 to June 1999, 83 patients affected by stomach cancer were accepted in our department: 12 patients (14.5%) were excluded from our study after the preoperative staging; in 71 patients (85.5%) an explorative laparoscopy as the first step of the operation was performed.

*3. Results* Laparoscopy confirmed preoperative staging in 53 cases (74.6%), in 12 patients demonstrated an overstaging. Laparoscopy demonstrated in 6 patients unsuspected causes of unresectability.

*4. Conclusions* When performed in patients affected by malignant neoplasm and declared resectable, intraoperative laparoscopy can demonstrate conditions not detectable by traditional preoperative investigations, consequently reducing to zero explorative laparotomies.


*Diagnostic and Therapeutic Endoscopy*, Vol. 6, pp. 125–131
Reprints available directly from the publisher
Photocopying permitted by license only

© 2000 OPA (Overseas Publishers Association) N.V.
Published by license under
the Harwood Academic Publishers imprint,
part of The Gordon and Breach Publishing Group.
Printed in Malaysia.

### Can Routine Laparoscopy Help to Reduce the Rate of Explorative Laparotomies for Gastric Cancer?

### Laparoscopy in Gastric Cancer

GIAN CARLO ROVIARO*, FEDERICO VAROLI, DAVIDE SONNINO, OMBRETTA NUCCA, GIANNI RABUGHINO and ALESSANDRO SCARDUELLI

*Department of General Surgery, University of Milan, Ospedale San Giuseppe, FBF, Via San Vittore 12, 20123 Milano, Italy*

*(Received 14 September 1999; Revised 1 November 1999; In final form 21 December 1999)*

**1. *Background* We developed this surgical protocol about performing intraoperative laparoscopy for staging in every patient affected by stomach cancer. Sensitivity and specificity of intraoperative laparoscopy are compared with conventional preoperative staging techniques.**

**2. *Methods* From January 1994 to June 1999, 83 patients affected by stomach cancer were accepted in our department: 12 patients (14.5%) were excluded from our study after the preoperative staging; in 71 patients (85.5%) an explorative laparoscopy as the first step of the operation was performed.**

**3. *Results* Laparoscopy confirmed preoperative staging in 53 cases (74.6%), in 12 patients demonstrated an overstaging. Laparoscopy demonstrated in 6 patients unsuspected causes of unresectability.**

**4. *Conclusions* When performed in patients affected by malignant neoplasm and declared resectable, intraoperative laparoscopy can demonstrate conditions not detectable by traditional preoperative investigations, consequently reducing to zero explorative laparotomies.**

*Keywords:* Explorative laparotomy, Gastric cancer, Laparoscopy, Staging of cancer

### INTRODUCTION

In the last few years, mini invasive surgery has completely changed surgical techniques, even for oncology. In order to complete staging and reduce the rate of explorative laparotomies, we performed explorative laparoscopy as the first step of the operation for every patient affected by stomach cancer who was considered resectable.

The staging of gastric cancer, performed with conventional preoperative diagnostic techniques, undoubtedly affects treatment and prognosis of this

* Corresponding author. Tel.: +39 02 8599 4799. Fax: +39 02 8599 4200.

disease. Infiltration of gastric serosa, peritoneal carcinosis, lymphonodal and hepatic metastases are valid prognostic factors, and they can remarkably modify the survival curves [1,2]. They represent the difference between a localized neoplasm, surgically resectable, and an advanced or diffuse disease.

When it has been indicated that an operation will be performed, laparotomy has always represented the traditional technique to ascertain and confirm the resectability of gastric cancer. To-date, using laparotomy, the interventions which only consist in an exploratory, bioptic or palliative phase, average between 15% and 35% following the different series, with a remarkable incidence of morbidity and mortality [3,4].

### MATERIALS AND METHODS

From January 1994 to June 1999, 83 patients affected by stomach cancer were accepted in our department: 52 males and 31 females. The average age was 68, ranging between 57 and 93. Preoperative staging consisted of an esophagogastric roentgenogram, esophagogastroscopy, abdominal echography, and computed tomography. In 55.5% of cases, neoplasms were localized in the antrum-pylorus, in 33.4% in the corpus-fundus, in 11.1% in the cardia.

Patients with preoperative evidence of carcinomatous ascites or pelvic, peritoneal and hepatic metastases were excluded from this series. Patients affected by a stenosating neoplasm, which would have anyway required a laparotomy for a palliative operation, were also excluded from the intraoperative laparoscopy protocol. Patients' anamnesis did not indicate previous surgical operations causing adhesive syndromes which could contra-indicate a laparoscopy.

### Laparoscopic Surgical Technique

In general anesthesia, the camera is introduced in the umbilical area, after creating pneumoperitoneum. We commonly employed 0° optics. Now we prefer to employ 30° optics, since their rotation allows for a wider visual field and a better vision especially of the subdiaphragmatic and pelvic areas. The choice of other trocars' position varies following patients' anatomy and their anatomicopathologic conditions.

Usually two 5 mm trocars are introduced in right pararectal and left flank, whose holes can be used for placing drains at the end of the operation. Liver, diaphragm, diaphragmatic pillars specifically for cardiac neoplasms, omentum, parietal and visceral peritoneum are inspected in order to detect the evidence of micrometastases or carcinomatosis of the omentum and peritoneum nondetected before (Fig. 1). The inferior side of hepatic lobes can be easily visualized by lifting them with dissectors or palpators (Fig. 2).

The possible local extent and the infiltration of gastric serosa is evaluated by direct vision and instrumental palpation (Fig. 3). Normally, gastric wall is soft and there is mobility between anterior and posterior wall. Conversely, areas infiltrated by the neoplasm are hard and rigid. The caudate lobe of the liver can be exposed by opening the gastrohepatic omentum and lifting the left hepatic lobe. Sliding the greater gastric curve to the left allows for visualization of the celiac lymphnodes and the possible infiltration of the superior margin of the pancreas.

The evaluation of the posterior gastric wall and its possible adherence to the retroperitoneum is performed by opening the gastrocolic ligament. Here, isolation of the vessels is performed with dissector and electrocoagulating scissors. Small vessels can be coagulated during dissection, and endoclips can be employed on other vessels. The stomach can be lifted in order to visualize the anterior face of the pancreas, the posterior gastric face and, in lean patients, the celiac lymphnodes. Next, the patient is placed in Trendelemburg position, and the optic is inverted to examine the pelvis. In women, the laparoscopic phase ends with the exploration of the ovary, in order to detect possible Krukenberg's localization. The average time of the procedure is 15 min (range: 12–45 min). The laparotomic phase can follow, if any condition of inoperability is detected.

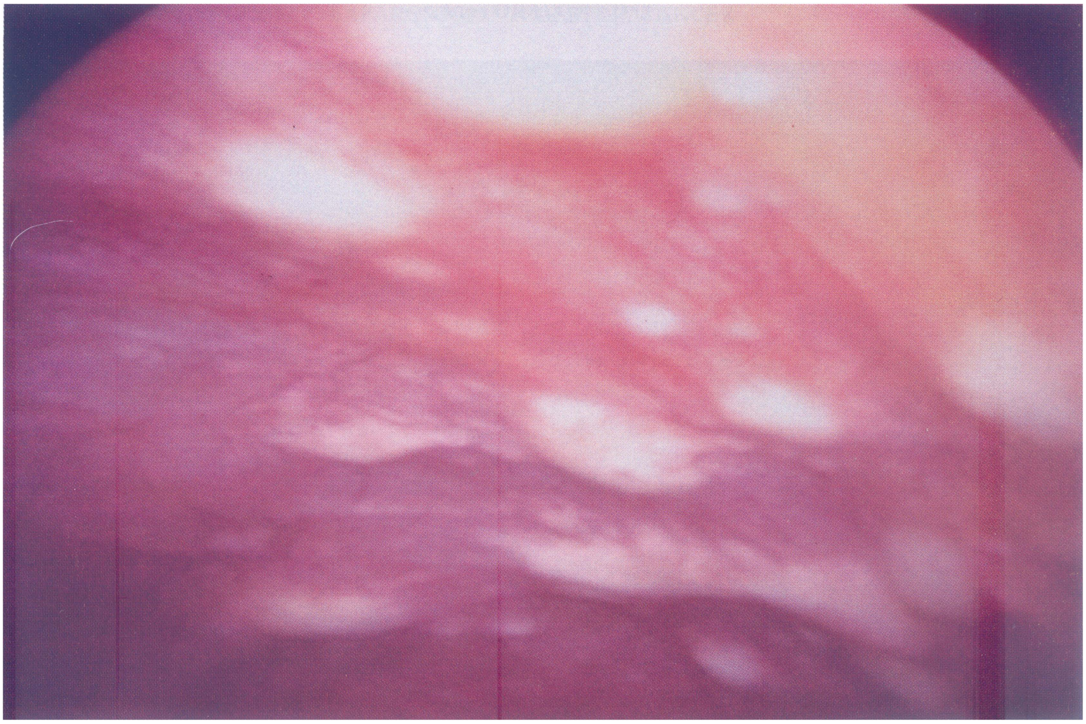

FIGURE 1 In this case intraoperative laparoscopic staging allowed the surgeon to discover peritoneal carcinosis undetected preoperatively, thus avoiding an unnecessary explorative laparotomy.

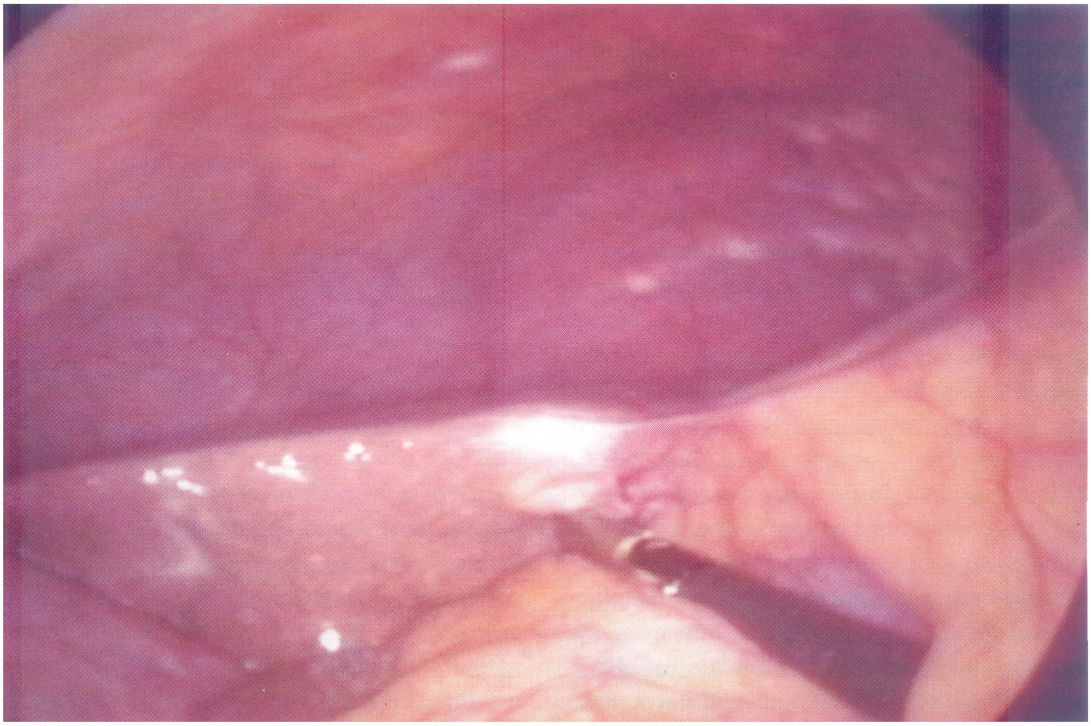

FIGURE 2 The inferior side of hepatic lobes can be easily visualized by lifting them with dissectors or palpators.

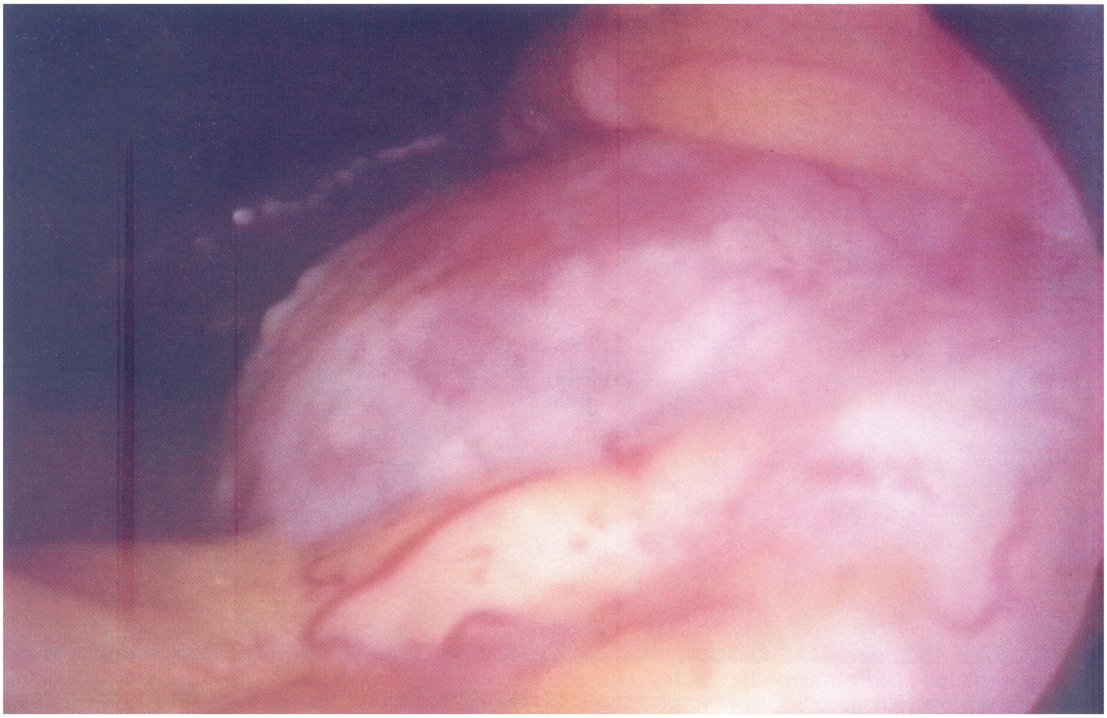

FIGURE 3 The possible local extent and the infiltration of gastric serosa is evaluated by direct vision and instrumental palpation.

### RESULTS

Out of 83 patients affected by gastric cancer, 12 were excluded from our study after preoperative staging. Out of these 12, three patients had distant metastases, one patient had carcinomatous ascites, and the remaining eight patients had a locally advanced but stenosating gastric neoplasm, which only required a palliative operation. Seventy-one patients (85.5%) were staged as a localized neoplasm and were consequently submitted to videolaparoscopic staging. Laparoscopy confirmed preoperative staging in 53 patients (74.6%).

Conversely, in 12 cases (16.9%), laparoscopy demonstrated a celiac and greater curve lymphonodal involvement preoperatively, but was not significant enough to cause inoperability. In 6 patients (8.5%) the laparoscopic phase revealed causes of inoperability, previously undetected, and thus avoided an unnecessary laparotomy. (Table I).

TABLE I Results

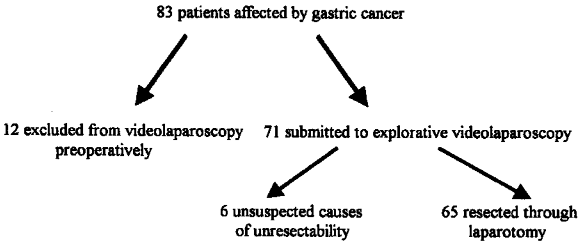

In 4 cases, a peritoneal carcinosis without ascites was evident: in two patients the neoplasm was located in the gastric antrum, and in the third patient was located in the cardia. A laparoscopic gastrostomy was performed only in the latter patient, afflicted by mild disphagia. In another patient, the neoplasm was infiltrating the serosa with invasion of the neighboring organs and a diffuse lymphonodal involvement. In the sixth patient, preoperative investigations had demonstrated a severe hepatic cirrhosis without esophageal varices: laparoscopy demonstrated a severe intraperitoneal portal hypertension and the infiltration by the neoplasm of the left hepatic lobe.

Among the patients submitted to laparotomy, laparoscopic data were confirmed in 64 patients (98.5%). In one case at the beginning of our experience we found a metastatic lymphoadenopathy at the celiac tripod undetected laparoscopically: however the surgical resection was performed with a R2 lymphectomy.

### DISCUSSION

An accurate preoperative staging is remarkably important both from a prognostic and therapeutic aspect. It determines a precise surgical option by a judgement of resectability of the lesion, and the choice of resection and the extent of lymphectomy. The evidence of hepatic and peritoneal metastases, lymphonodal involvement and loco-regional extent of the neoplasm are key factors to determine the surgical indication of resectability. These factors can cause statistically significant differences in survival rates after 5 years [2,4,5]. Additionally, infiltration of the gastric wall layers is responsible for decreasing the survival rate from 94% when only the mucosa is involved, to 5–10% when the serosa is infiltrated [1]. Many papers in scientific literature describe sensitivity, specificity and accuracy of echography and computed tomography (CT) [6–12].

Several authors associated diagnostic laparoscopy with these investigations as a completion of the preoperative staging. First oncological laparoscopic studies date to 1938 [13], but comparison between different diagnostic techniques dates to last twenty years, when the exam was mainly performed with local anaesthesia. The specificity of laparoscopy has always been high, as demonstrated by Possik in 1986, Watt and Steward in 1989 and Kriplani in 1991 [12,14,15]. The accuracy of echography regarding hepatic metastases in several series is 80–93%, reaching 97%. This accuracy is lower for CT, with values of 80–85% [6–12]. This parameter is directly proportional to diameter of the metastasis: diameters between 1 and 3 cm can be easily missed by CT [11], as demonstrated by laparotomic and necroscopic evidences.

Laparoscopy reaches accuracy of 96–96.5% [14,16] affected by hepatic metastases, demonstrated no false positives with laparoscopy, and false negative were only 3%. Conversely, false negatives were 13% for echography and 11% for CT. Additionally, laparoscopy had an accuracy of 88.5% in diagnosis of infiltration of gastric serosa (T3), and 81.2% in diagnosis of invasion of neighboring structures (T4). The evidence of peritoneal metastases, absolute contra-indication to a major surgical resection, is detected with laparoscopy with an accuracy ranging between 89.4% and 98%, with false negatives of 0–1%, versus 7–8% for echography and 9% for CT. There are no significant differences between instrumental investigations regarding lymphonodal involvement.

Thus, considering main parameters affecting resectability and prognosis of gastric cancer, laparoscopy happens to be the most reliable investigation in order to confirm the positivity of pathologic findings detected, with a percentage of false negatives around zero. Notwithstanding, laparoscopy has never been routinely employed for staging, and the majority of studies about it were carried on in gastro-enterologic divisions, where the exam was employed as preoperative investigation.

The age of mini invasive surgery opened new perspectives in every field of traditional surgery, and also intraoperative staging has been involved. Based on these aspects and on our experience of more than 3000 videolaparoscopic operations in 5 years, we developed this surgical protocol about performing intraoperative laparoscopy in every patient affected by malignant neoplasm as the first step of the surgical operation. Undoubtedly, the most important result of our study is the percentage of patients declared resectable with echography and CT, which on the contrary were demonstrated inoperable by laparoscopy (7% of cases). Thus, an unnecessary laparotomy was avoided.

Before 1994, our series regarding surgical treatment of gastric cancer reported an explorative laparotomies rate of 7.5%. This rate, even if lower than in other series, has been reduced to zero by standardized use of intraoperative laparoscopy. Undoubtedly, this aspect cannot affect the survival rate, but greatly improved the clinical course of these patients, abolishing explorative laparotomies in our experience.

The same situation happened in our experience for lung cancer: in our department every resectable patient is submitted to videothoracoscopic operative staging (VOS) [17]. Out of 446 patients affected by lung cancer, thoracoscopy demonstrated unsuspected conditions of inoperability in 22 cases, avoiding unnecessary thoracotomies. In this way, the rate of explorative thoracotomies is 2.8%, mainly due to cases at the beginning of our experience or to adhesive conditions where the procedure was impossible to perform.

Specifically regarding gastric cancer, intraoperative laparoscopy can provide important data regarding regional lymphnodes, particularly about the retroperitoneal ones. However, the exploration of the omental bursa can be very difficult when abundant fatty tissue or post-inflammatory gastropancreatic adhesions hinder the visualization of this area.

Literature has already demonstrated that patients affected by a locally advanced disease, surgically resectable, have low possibility of cure after surgery followed by chemotherapy. In fact, an increasing number of therapeutic protocols recommend a neoadjuvant treatment for these patients, provided that they are accurately staged, especially regarding regional lymphnodes N2 [18,19]. We recorded only one case of N2 lymphonodal involvement of celiac nodes, missed by laparoscopy and discovered intraoperatively: this happened at the very beginning of our experience when our technical ability was lesser. Viceversa laparoscopy demonstrated the evidence of N2 lymphnodes not detected by preoperative staging in 12 cases (16.9%): but, since a neoadjuvant protocol had not already been planned, we decided not to postpone the operation.

Intraoperative laparoscopy must be considered as a real surgical procedure, requiring a surgical training in order to make the procedure accurate and easy. Intraoperative laparoscopy demonstrates unquestionable advantages for a precise staging of gastric cancer and a correct surgical option. It is an easy technique, with a slight modification on the operation global time, and free of mortality and morbidity. When performed in every patient affected by malignant neoplasm and declared resectable, intraoperative laparoscopy can demonstrate conditions of diffuse pathology not detectable by traditional preoperative investigations, consequently reducing to zero explorative laparotomies.

### *References*

[1] Boku, T., Nakane, Y. *et al.* Prognostic significance of serosal invasion and free intraperitoneal cancer cells in gastric cancer. *Br. J. Surg.* 1990; **77**: 436.

[2] Bonekamp, J.J., Van der Velde, C.J.K. and Kampscoer, G.H.M. Comparison of factors influencing the prognosis of Japanese, German and Dutch gastric cancer patients. *World J. Surg.* 1993; **17**: 410–415.

[3] Wanebo, H.J., Kennedy, B.J., Chmiel, J. *et al.* Cancer of the stomach: a patient care study by the American College of Surgeons. *Ann. Surg.* 1993; **218**: 583–593.

[4] Breaux, G.R., Bringaze, W. and Chappuis, C. Adenocarcinoma of the stomach: a review of 35 years and 1710 cases. *World J. Surg.* 1990; **14**: 580–586.

[5] Maruyama, K. The most important factors for gastric cancer patients. A study using univariate and multivariate analyses. *Scand. J. Gastroent.* 1987; **22**: 63–68.

[6] Barth, R.A., Jeffrey, R.B., Moss, A.A. *et al.* A comparison study of computed tomography and laparoscopy in the staging of abdominal neoplasms. *Dig. Dis. Sci.* 1981; **26**: 253–256.

[7] Boyd, W.P. Relative diagnostic accuracy of laparoscopy and liver scanning techniques. *Gastrointest. Endosc.* 1982; **28**: 104–106.

[8] Kemeny, M.M., Sugarbaker, P.H., Smith, T.J. *et al.* A prospective analysis of laboratory tests and imaging studies to detect hepatic lesions. *Ann. Surg.* 1982; **195**: 163–167.

[9] Kleinhaus, U. and Militianu, D. Computed tomography in the preoperative evaluation of gastric carcinoma. *Gastroin. Radiol.* 1988; **13**: 97–101.

[10] Lamb, G. and Taylor, I. An assessment of ultrasound scanning in the recognition of colorectal liver metastases. *Ann. R. Coll. Surg. Engl.* 1982; **64**: 391–393.

[11] Smith, T.J., Kemeny, M.M., Sugarbaker, P.H. *et al.* A prospective study of hepatic imaging in the detection of metastatic disease. *Ann. Surg.* 1982; **195**: 486–491.

[12] Watt, I., Stewart, I. and Anderson, D. Laparoscopy, ultrasound and computed tomography in cancer of oesophagus and gastric cardia: a prospective comparison for detecting intra-abdominal metastases. *Br. J. Surg.* 1989; **76**: 1036–1043.

[13] Benedict, E.B. Peritoneoscopy. *N. Engl. J. Med.* 1938; **218**: 713–714.

[14] Possik, R.A., Franco, E.I., Pires, D.E. *et al.* Sensitivity, specificity and predictive value of laparoscopy for the staging of gastric cancer and for the detection of liver metastases. *Cancer* 1986; **58**: 1–6.

[15] Kriplani, A.K. and Kapur, B.M.L. Laparoscopy for preoperative staging and assessment of operability in gastric carcinoma. *Gastroint. Endosc.* 1991; **37**(4): 441–443.

[16] Shandall, A. and Johnson, C. Laparoscopy or scanning in oesophageal and gastric carcinoma? *Br. J. Surg.* 1985; **72**: 449–451.

[17] Roviaro, G.C., Varoli, F., Rebuffat, C. *et al.* Videothoracoscopic staging and treatment of lung cancer. *Ann. Thorac. Surg.* 1995; **59**: 971–974.

[18] Ajani, J.A., Mansfield, P.F. and Ota, D.M. Potentially resectable gastric carcinoma: current approaches to staging and preoperative therapy. *World J. Surg.* 1995; **19**: 216–220.

[19] Kelsen, D.P. Adjuvant and neoadjuvant therapy for gastric cancer. *Semin. Oncol.* 1996; **23**(3): 379–389.

